# Accumulation of CD45RO+CD8+ T cells is a diagnostic and prognostic biomarker for clear cell renal cell carcinoma

**DOI:** 10.18632/aging.203045

**Published:** 2021-05-19

**Authors:** Ke Wu, Xinyi Zheng, Zhixian Yao, Zhong Zheng, Wenjie Huang, Xingyu Mu, Feng Sun, Zhihong Liu, Junhua Zheng

**Affiliations:** 1 Department of Urology, Shanghai General Hospital, Shanghai Jiao Tong University School of Medicine, Shanghai 200080, China; 2 Department of Pharmacy, Huashan Hospital, Fudan University, Shanghai 200040, China

**Keywords:** clear cell renal cell carcinoma, CD45RO+CD8+ T cells, biomarker, UCHL1

## Abstract

Renal cell carcinoma is characterized by high immunogenicity and infiltration of immune cells. CD45RO+CD8+ T cells are well known as a critical role in host defense of the immune environment. However, their role in clear cell renal carcinoma (ccRCC) remains unknown. To elucidate the clinical importance of CD45RO+CD8+ T cells in ccRCC as well as its underlying mechanism, we analyzed several types of peripheral immune cells from 274 patients with ccRCC who have received radical or partial nephrectomy and 350 healthy people. Flow cytomety assays showed there was no significant difference in the proportions of CD8+ T cells and its subtypes other than CD45RO+/CD45RA+CD8+ cells. Both gene and protein expression levels of CD45RO in ccRCC tissues were decreased. CD45RO+CD8+ T cells showed increased proliferative abilities but decreased apoptotic abilities through MAPK signaling activation in ccRCC. High expression level of CD45RO+CD8+ T cells inhibited ccRCC progression, including proliferation, invasion, as well as autophagy of ccRCC through many signaling pathways. Bioinformatics and immunohistochemical chip analysis measured gene and protein levels of CD45RO and other related proteins. The combination of UCHL1, HMGB3, and CD36 has diagnostic value in ccRCC and is able to predict prognosis. Collectively, CD45RO+CD8+ T cells play a critical role in ccRCC progression and may be regarded as clinical indicators.

## INTRODUCTION

Renal cell carcinoma (RCC) is the ninth most common malignant disease worldwide [1]. Histologically, clear cell renal cell carcinoma (ccRCC) constitutes 70% of RCC. Neoplasms deriving from the kidney, including the renal pelvis, are evaluated to occur in more than 60000 patients and resulted in more than 20000 deaths in China [2]. Furthermore, the incidence rate has progressively escalated, particularly in young patients or those with high-grade disease [3]. Although the morbidity has decreased with the adoption of better treatment procedures, including laparoscopic, robotic operations, chemotherapy, radiotherapy and tumor immune therapy, the lack of early diagnostic and prognosis indicators for ccRCC leads to the prognosis remaining desperately poor [4].

RCC is characterized by great high immunogenicity, with a tremendous infiltration of immune cells, notably in T cells, NK cells, dendritic cells, macrophages and so on [5]. Remarkably, therapies targeting tumor immune microenvironment such as non-specific immunomodulators, vaccine gene therapy, monoclonal antibodies, colony stimulating factors, interleukins, and interferons have become the mainstays for the immune treatment of RCC in recent years [6]. Nowadays, with the growing understanding of escape mechanisms over the last decade, significant improvements have been made. Immune-checkpoint blockades (ICBs) including the well-known PD-1/PD-L1 as well as CTLA-4 have produced durable response rates and survival improvement in RCC and monoclonal antibodies such as nivolumab, pembrolizumab, atezolizumab and avelumab are being tested in RCC [7]. Taken together, researchers tried to activate T cells and weaken tumor immune escape in immune environment [8]. Considering the significance of the anti-tumor response by CD8+ T lymphocytes, not merely the efficacy of T cells matters, the component especially the proportionality of mature T cells activated by MHC and co-stimulator should be focused on [9]. It is critical to exploit the compositions of T lymphocytes and explore their clinical importance deeply [10]. Markers, such as CD45, CD28 and CD25, representing status and function of CD8+ T cell are well worthy of further study.

CD45, the leukocyte common antigen (LCA) known at first, belongs to sort of protein tyrosine phosphatase, receptor type C, encoded by PTPRC in leukocyte signaling controlling cell growth, differentiation, mitosis and oncogenic transformation [11]. The present of various CD45 isoforms is cell type dependent and relies on the phase of stimulation of cells and phase of differentiation [12]. As for homosapiens, CD45RO and CD45RA are considered as memory and naïve T cells in respective and T cell receptors (TCR), furthermore, are directly mediated by CD45 in cell signaling pathway [13]. It is well-known that memory T lymphocytes originate and develop during cell-regulated immunological responses and exist for a long spectrum of time, typically years even the antigens are eradicated from body [14]. These memory-specializing T lymphocytes take responsibilities for swifter and enhance responses to the secondary along with exposures of subsequence to specific antigens, which stands that durable T cells have indispensable significant role to play in host adaptive defense for infection [15]. However, their anti-tumor functions in humans cannot be ignored. A recent study about colorectal cancer has revealed that CD45RO is more associated with increased survival, and less early metastasis [16]. Nevertheless, CD45RA, in acute myeloid leukemia, is strongly correlated with leukemic stem cells which, could be responsible for relapses after therapy in acute myeloid leukemia [17]. Another study reported that CD45RO is lower yet CD45RA is higher in peripheral T cell lymphoma. However, even if those findings precisely depicted that both memory and naive leukocytes play significant roles in tumor-associated immunity, the underlying mutuality and association between CD45RO/CD45RA T cells and RCC are still hiding in the shadow [18].

Hence, in this article, we managed to exploit the clinical importance of CD45 in ccRCC by analyzing proportion changes of CD45 subtypes and discover the diagnostic and prognosis significance of CD45 subtypes combined with other related immune indicators to unveil novel targets for ccRCC.

## MATERIALS AND METHODS

### Specimen and patients

The Ethics Committee of Shanghai General Hospital approved this study. The samples were all obtained with informed consent from patients with ccRCC and healthy people during June 2010 and June 2018. Peripheral blood (274 ccRCC patients and 350 healthy people) and tissues (35 ccRCC tissues and 35 adjacent normal tissues in correspondence) were collected and obtained from Shanghai General Hospital, Shanghai Jiao Tong University School of Medicine. The inclusion criteria in this study were as follow: 1) new ccRCC cases were diagnosed by clinicopathological examinations; 2) no patients have undergone the radiotherapy or chemotherapy; 3) patients agreed to the donation of tissues and recruited in a 5-year clinical follow-up visit. The exclusion criteria in this study were as follow: 1) the patients who have taken relevant treatments before admission; 2) the patients bearing other clinical diseases or disorders. All research abided by the principles of the Declaration of Helsinki. This study was approved by the Research Ethics Committee of Shanghai Jiaotong University and informed consent was obtained.

### Flow cytometry

We extracted peripheral blood mononuclear cells (PBMCs) by utilizing lymphoprep gradient density centrifugation (AS1114544, Axis-Shield, Norway). PE anti-human CD45RO (304206, Biolegend, USA), APC anti-human CD45RA (304112, Biolegend), FITC anti-human CD8 (100706, Biolegend), PE-cy5 anti-human CD4 (CD45-T2-100, Alpha Diagnostic International), Alexa Fluor^®^ 488 anti-human CD25(356115, Biolegend) and PerCP anti-human CD3(344813, Biolegend) were used to stain PBMCs, which are co-cultured with OSRC-2 for 48 hours. CD45RO+CD8+ T lymphocytes and CD45RA+CD8+ T lymphocytes were extracted by BD FACSAria II (Becton Dickinson, USA). The fineness of all extracted PBMCs conventionally surpassed 90%. We assembled and suspended 1×10^6^ cells in phosphate-buffered saline (PBS) and then cultured for 5 min at 4°C. Subsequently, the isolated cells were cultured on crushed ice for 0.5 hour in 50 μl of staining solution with 1 μg/ml of corresponding conjugated antibodies. The stained cells were rinsed twice with the pre-cooling PBS. All the samples were examined utilizing the FACS Calibur flow cytometer (BD) and data were analyzed utilizing CellQuest software (BD).

### Immunochemistry

After being fixed and decalcified, the blocks were processed into routine slices and then underwent dewaxing with xylene and hydration with gradient alcohol. The slices were applied with 3% H2O2, followed by 15-min resting to blockade endogenous peroxidase activities remaining in tissues, and then were washed with PBS triply. The ccRCC slices were applied with the citrate solution (10 mM) and heated in microwave oven for 20 min for antigen retrieval. The ccRCC slices were subsequently applied with 5% serum of goat for 10 min, for the sake of blocking the nonspecific binding. After applying with the primary antibodies of CD8 (1/200, ab4055, Abcam, UK), CD45RO (5 μg/ml, ab23, Abcam), HMGB3 (1/100, ab75782, Abcam), IKBKE (10 μg/ml, ab7891, Abcam), PLTP (1/50, ab189776, Abcam), PTGES (10 μg/ml, ab62050, Abcam) and CD36(1:100 ab133625, Abcam), the slices were preserved at 37°C for 0.5 hour and rinsed with PBS. After applying with the secondary antibody (18653, Cell Signaling Technologies, USA) labelled horseradish peroxidase, the slices were preserved at 37°C for 0.5 hour and rinsed with PBS once again, stained utilizing the diaminobenzidine (DAB) (91-95-2, Sigma-Aldrich Chemical Company, USA) for 10 min, rinsed with running purified water, stained with hematoxylin, added with neutral resins and eventually observed utilizing the microscope and the pictures were taken.

### Cell culture

The RCC cell line, OSRC-2 (human), Caki-2 (human) and RAG (mice) were originally purchased from National Collection of Authenticated Cell Cultures (China). OSRC-2 was cultured in DMEM (Invitrogen, USA) added with fetal bovine serum (Gibco, USA) of 10% in the cell incubator with 5% CO_2_ environment and humidification at 37°C. For immobilized T cell activation, CD3/CD28 TAC soluble activator (Sigma, USA) was utilized in accordance with the manufacturer’s protocol.

### Mouse model of orthotopic tumor xenograft

OSRC-2 cells were applied at 2 × 10^6^ cells per mouse, into the sub-capsule of left kidneys in 6-week-old BALB/c male nude mice (Shanghai Model Organisms Center, China) (*n* = 10 mice/group). For adoptive transfer experiment, CD45RA+CD8+ T lymphocytes and CD45RO+CD8+ T lymphocytes were extracted from the bone marrow (BM) of wild type (WT) mice by Flow cytometry isolation and were transferred to each experimental mouse with 5 × 10^6^ cells (one times per 2 days for 2 weeks). Tumor volume and body weight of each mouse were examined, and survival time were recorded. Tumor bulk was analyzed by the following formula: volume = 1/2 × length × (width)^2^. The animal study was scrutinized and approved by The Institutional Review Board of Shanghai Jiaotong University.

### Western blotting

Grinding adjacent normal tissues and ccRCC tissues were washed in PBS, collected with a cell scraper and ultracentrifuged for 20 min at 10,000 × g at 4°C. The sediments were resuspended in high efficiency cell tissue rapid lysis buffer (RIPA; P0013J, Beyotime, China) with 1% phenylmethanesulfonylfluoride proteinase inhibitors (PMSF; P1008, Beyotime) and 1% phosphatase inhibitors (C071, ChemDiv, USA) inside RIPA. Subsequently, the mixtures were boiled at 95°C for 10 min and then were preserved at −80°C if necessary. Protein quantification were implemented by utilizing the BCA protein assay kit (P0012, Beyotime, China). Total protein mixtures of 20 μg were electrophoresed by direct current in SDS-PAGE gels (LK102, Epizyme Biotech, China) using a Miniprotein III machine (Bio-Rad, USA) and were electro-transferred to PVDF membranes (Millipore, USA) for 2 h, followed by overnight application with primary antibodies targeted CD45RO (5 μg/ml; ab23, Abcam) and GAPDH (1/10000; ab181602, Abcam) at 4°C. Then the membranes were rinsed in triplicates with PBST solution (P0222, Beyotime) and were applied with the secondary goat anti-rabbit IgG antibodies (1:5000; Bioworld Technology, USA) at 20°C for 1 h. Subsequently, the membranes were rinsed again in triplicates and prepared for chemiluminescence by utilizing the Immobilon Western Chemiluminescent HRP Substrate Kit (Millipore).

### Real-time quantitative polymerase chain reaction (RT-qPCR)

For the detection of transcriptome alteration in ccRCC, total RNA, including miRNA, was obtained by RNAsimple Total RNA kit (DP419, Tiangen, China) from cells and tissues. cDNA preparation of mRNA samples was implemented by QuantScript RT Kit (KR201, Tiangen, China). All cDNA were treated by Quant one step qRT-PCR Kit (SYBR-Green) (FP303, Tiangen, China). The thermocycling criteria were as follow: 1 cycle at 50°C for 30 mins; 1 cycle at 95°C for 2 mins; 40 cycles at 94°C, 55°C and 68°C for 20 secs. For the sake of RNA normalization, primers of GADPH were utilized. To examine the relative transcript levels of UCHL1, the 2^−ΔΔCq^ method was applied. All primers were obtained from Shangon Company (China). The sequences of relevant primers were shown as follow: 5′- ATAGCCAGCATCAGTTGCCT -3′ (Forward) and 5′- TCGTCTACCAACCTTTGCCC -3′ (Reverse) for UCHL1; and 5′- GCAACTAGGATGGTGTGGCT -3′ (sense) and 5′- TCCCATTCCCCAGCTCTCATA -3′ (antisense) for GAPDH.

### Bioinformatics

Gene Set Variation Analysis (GSVA), a algorithm that evaluates variation of biological pathways over a sample population based on single sample GSEA (ssGSEA) score. We utilized median expression value of UCHL1 to divide TCGA RCC patients into 2 groups: UCHL1 high and UCHL1 low. GSVA analysis was conducted to acquire the score of pathways among all TCGA RCC samples. Limma, a R package were applied to detect differentially expressed pathways (DEPs) between 2 groups. Hierarchical clustering was applied on oncogenic DEPs on the heatmap. 988 immune response related genes in human were downloaded from InnateDB [3] to further explore the highly related ones with UCHL1.

### Gene array

After co-cultured with OSRC-2, CD8+ T lymphocytes (*n* = 10) were extracted and characterized by Affymetrix GeneChip^®^ Human Gene 2.0 ST Array. RNA quantification was implemented by NanoDrop 2000 spectrophotometer (Thermo Fisher Scientific, USA) by detecting the absorbance at 260 nm with quality control standards being A260:A280 = 1.8–2.1. RNA was tagged utilizing FlashTag^®^ Biotin HSR labeling kit (Genisphere, HSR30FTA) in accordance with the instructions of manufacturer. Comprised of 1.35 million distinct probes (25-mers) in Each Human Gene 2.0 ST Array and represented by 21 probes (median) in each transcript, the statistical confidence was robustly enhanced. In order to identify individual transcripts precisely, each transcript is identified by a specific intron or exon probe.

### Statistics

Statistical analysis was applied by utilizing Prism 8 (GraphPad Software, USA). All data were shown as means±SEM and examined for differences between disparate control and treatment groups utilizing two-way ANOVA or Student’s *t*-test (two-tailed). Correlation significance between parameters was examined by utilizing linear regression. *P* values ≤0.05 in differences were reckoned significant.

### Data availability statement

All datasets presented in this study are included in the article.

### Ethics statement

The protocol was approved by Shanghai General Hospital.

## RESULTS

### CD8+ T cells of ccRCC are positively correlated with tumor grade and contribute to patient prognosis

**Figure 1:**
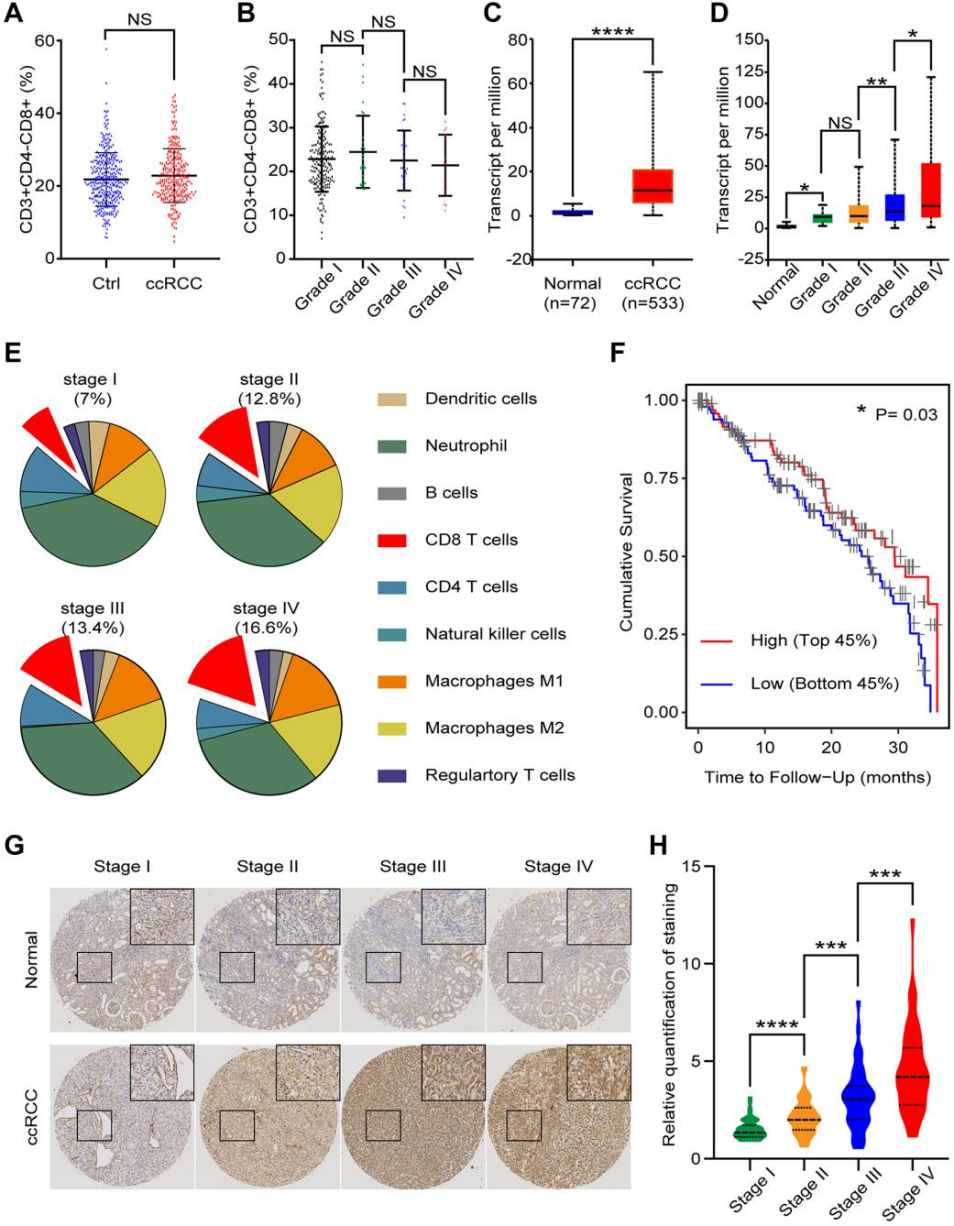
**CD8+ T cells in peripheral blood and tissues of ccRCC patients.** We analyzed clinical and bioinformatics data of CD8+ T cells. (**A**) Proportion of CD8+ T cells (CD3+CD4-CD8+) in CD3+ cells of peripheral blood from healthy people (*n* = 350) and ccRCC patients (*n* = 274). (**B**) Proportion of CD8+ T cells in peripheral CD3+ cells of different grades of ccRCC (grade I: *n* = 198; grade II: *n* = 28; grade III: *n* = 32; grade IV: *n* = 16). (**C**) *CD8a* transcript level of ccRCC tissues (*n* = 533) and normal renal tissues (*n* = 72) according to TCGA database. (**D**) *CD8a* transcript level of different grades ccRCC (normal: *n* = 72; grade I: *n* = 14; grade II: *n* = 229; grade III: *n* = 206; grade IV: *n* = 76). (**E**) According to TCIA database, proportion of immune cells in different grades of ccRCC tissues. (**F**) Cumulative survival time of patients with high level of CD8+ T cells and low level of CD8+ T cells. (**G**) Representative IHC image of different stage of ccRCC tissues. (**H**) Relative quantification of staining of ccRCC tissues. Means ± SEM of experiment performed in triplicates are shown. ^*^P < 0.05; ^**^p < 0.01; ^****^p < 0.001; NS, not significant.

To assess the potential immune differences between healthy people (*n* = 350) and ccRCC patients (*n* = 274), we collected peripheral blood and tested CD8+ T cells (CD3+CD4−CD8+) proportion of CD3+ cells. The flow cytometry results showed that none of statistically significant differences (Figure 1A). We clarified ccRCC patients into 4 grades (I~IV) in accordance with the grading classification of World Health Organization (WHO) and International Society of Urological Pathology (ISUP). Further analysis found that no significant differences of CD8+ T cells proportion among patients with different grades of ccRCC (grade I: *n* = 198; grade II: *n* = 28; grade III: *n* = 32; grade IV: *n* = 16) (Figure 1B). TCGA database collected genetic information of normal renal tissues and ccRCC tissues. We analyzed these data and found inconsistent result compared to our peripheral blood data. We found that *CD8a* (Gene of CD8) transcript level of ccRCC tissues (*n* = 533) were significantly higher than normal kidney tissues (*n* = 72) (Figure 1C). Further analysis showed that there was a difference of *CD8a* transcript level among different grades of ccRCC (normal: *n* = 72; grade I: *n* = 14; grade II: *n* = 229; grade III: *n* = 206; grade IV: *n* = 76) (Figure 1D). These results elucidated that proportion of CD8 T lymphocytes in ccRCC immune environment maybe higher and increased with tumor grade.

ccRCC tissues contain a variety of immune cells, including monocytes (M1 and M2 macrophages), lymphocyte (B cells, T cells and NK cells), and neutrophil etc. According to The Cancer Immune Atlas (TCIA, https://tcia.at/home) database, immunological composition analysis indicated that CD8 T cells gradually increase with ccRCC stages (stage I:7%; stage II:12.8%; stage III:13.4; stage IV:16.6%) (Figure 1E). To explore the clinical implication of CD8+ T cells in ccRCC, we divided ccRCC patients of TCGA into two groups (High CD8+ T lymphocytes: Top 45% and Low CD8+ T lymphocytes: Bottom 45%). Cumulative survival time indicated that the prognosis of ccRCC patients with high level of CD8+ T lymphocytes were better than ccRCC patients with low level of CD8+ T lymphocytes (*p* = 0.0300) (Figure 1F). To verify these bioinformatics analysis results, we then performed CD8 immunohistochemical staining of normal renal tissues, low grade ccRCC tissues and high grade ccRCC tissues (Figure 1G). The quantitative analysis results showed that CD8 was expressed in higher level in ccRCC tissues than the normal ones, especially in high grade ccRCC tissues (Figure 1H). In genenral, these results implecated that there was an association between CD8+ T lymphocytes and ccRCC progression: CD8+ T lymphocytes gradually increased with ccRCC progression, while for patients with the similar grade, CD8+ T cells were good for prognosis.

### Differences of CD8^+^ T lymphocytes subsets between the healthy and the ccRCC

**Figure 2:**
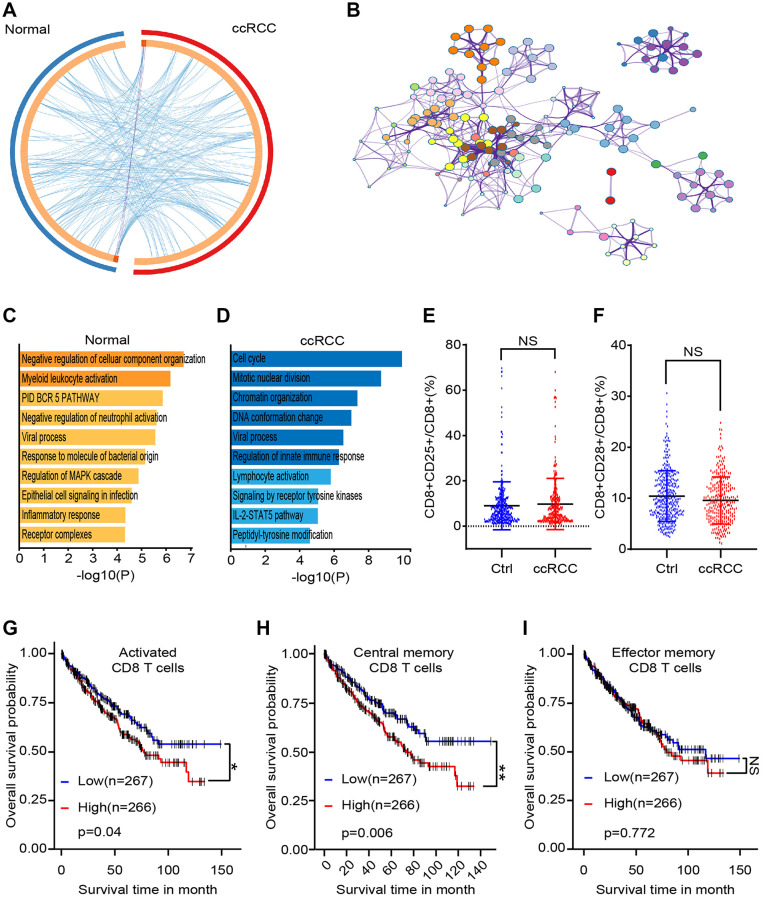
**Differences between CD8+ T cells subsets of healthy people and ccRCC patients.** We collected clinical data and bioinformatics data of peripheral CD8+ T cells and analyzed differences. (**A**) Gene chip tested differential genes of CD8+ T cells from peripheral blood of 5 healthy people and 5 ccRCC patients. (**B**) Pathways enrichment of different gene between healthy group and ccRCC group. (**C**, **D**) Differential gene enriched pathways of CD8+ T cells after co-cultured with/without RAG cells. (**E**, **F**) Proportion of CD8+CD25+ T cells (E) and CD8+CD28+ T cells of CD8+ T cells from healthy people (*n* = 350) and ccRCC patients (*n* = 274) (F). (**G**, **H**, **I**) Survival time of ccRCC patients with different level of activated CD8+ T cells (CD25+CD8+ T cells), activated CD8+ T cells (CD25+CD8+ T cells) and effector memory CD8+ T cells (CD45RO+CD62L-CD8+ T cells). Means ± SEM of experiment performed in triplicates are shown. ^*^P < 0.05; ^**^p < 0.005; ^***^p < 0.005; ns, not significant.

To explore the potential changes of CD8+ T lymphocytes in ccRCC patients, we obtained peripheral blood samples from 5 healthy people and 5 ccRCC patients. CD8+ T lymphocytes were sorted by flow cytometric and differential genes were analyzed by gene chip before and after co-culturing. There were only 3 common differential genes between these two groups (log|FC|> 2, *p* < 0.05) (Figure 2A). To simulated biological function of differential genes, we purified peripheral blood mononuclear cells of BALB/c mice and co-cultured with/without RAG cells (renal carcinoma cell line from BALB/c mice) for 48 hours. We analyzed different genes of CD8+ T cells and cluster analysis showed that the pathways for gene enrichment were basically inconsistent (Figure 2B, 2C). We analyzed differential genes of CD8+ T cells, which were accumulated in several signaling pathways (Figure 2D). The gene enriched signaling pathways suggested that there were statistical differences in CD8+ T cells, which may be caused by different cell subpopulations. To verify our hypothesis, we compared peripheral activated CD8+ T lymphocytes (CD8+CD25+ T lymphocytes) of healthy people with ccRCC patients and found that there are no significant differences (Figure 2E). It has been studied that the equilibrium between CD8+CD28+ T lymphocytes and CD8+CD28- T lymphocytes are significant in the mechanisms of pathogenesis. The result showed that there were no statistical differences of CD8+CD28+ T lymphocytes was found between healthy people and ccRCC patients (Figure 2F). As for ccRCC immune environment, activated CD8+ T lymphocytes (CD25+CD8+ T lymphocytes) (*p* = 0.0398) (Figure 2G) and central memory CD8+ T cells (CD45RO+CD62L+CD8+ T cells) (*p* = 0.0057) (Figure 2H) but effector memory CD8+ T cells (CD45RO+CD62L-CD8+ T lymphocytes) (*p* =0.77165) (Figure 2I) indicated worse prognosis of ccRCC patients. These results illustrated that immune status of CD8+ T lymphocytes were potential differences between healthy people and ccRCC patients.

### CD45RO+CD8+ T lymphocytes decreased in ccRCC tissues

**Figure 3:**
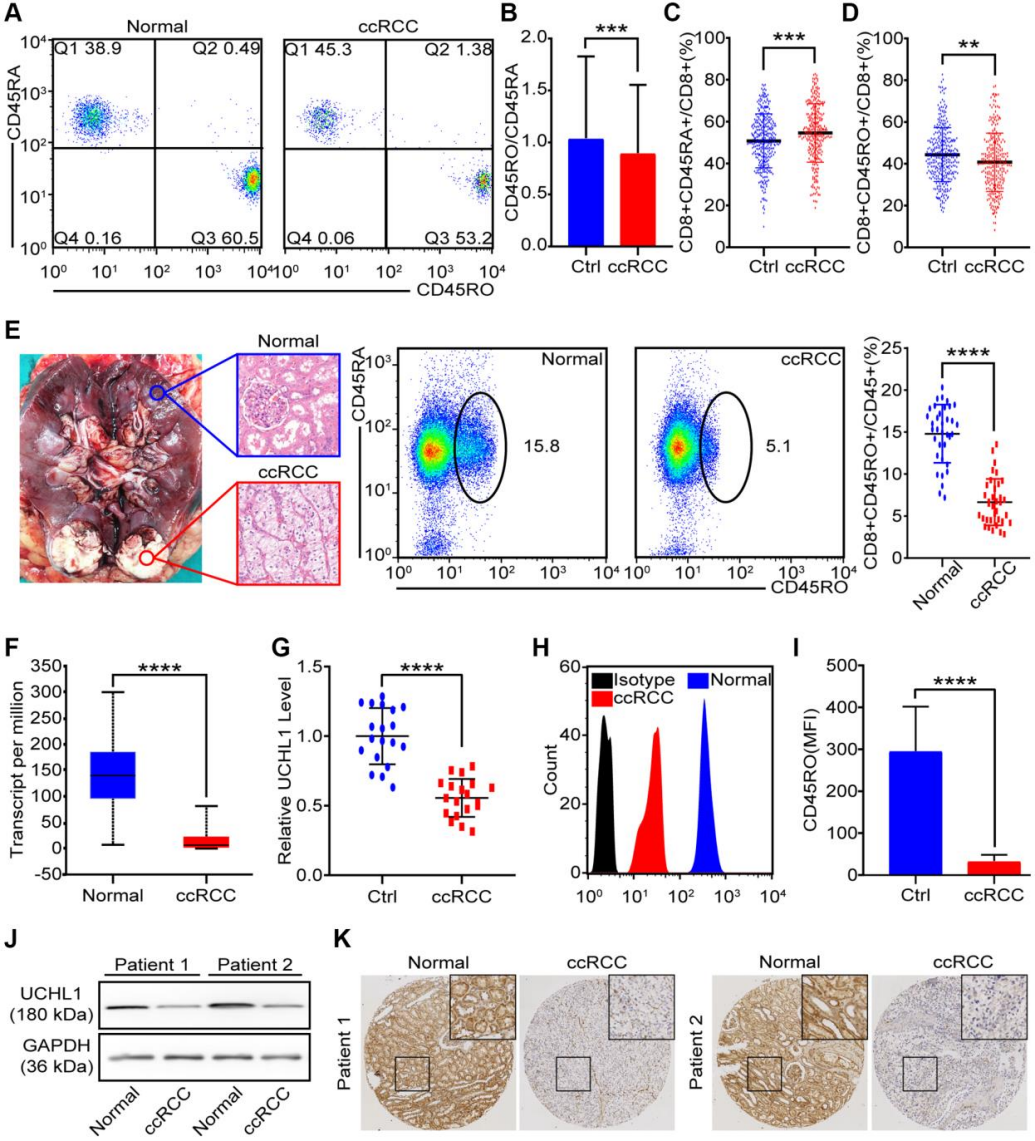
**CD45RO+CD8+ T cells decreased in ccRCC tissues.** We collected PBMCs and renal tissues to analysis proportion of CD45RO+CD8+ T cells/CD45RA+CD8+ T cells. (**A**) Flow cytometry representative image of CD45RO+CD8+ T cells/CD45RA+CD8+ T cells from healthy people and ccRCC patients. (**B**, **C**) Proportion of CD45RA+CD8+T cells (B) and CD45RO^+^CD8^+^ T cells (C) of CD8^+^ T cells from healthy people and ccRCC patients. (**D**) Ratio of CD45RA+CD8+ T cells and CD45RO+CD8+ T cells in peripheral blood of healthy people and ccRCC patients. (**E**) Pathologically confirmed adjacent normal renal tissues (*n* = 35) and ccRCC tissues (*n* = 35) were collected and flow cytometry was used to test proportion of CD45RO+CD8+ T cells. (**F**) Transcript level of *UCHL1* (gene name of CD45RO) in normal renal tissues and ccRCC tissues according to TCGA database. (**G**) Transcript level of *UCHL1* in adjacent normal renal tissues (*n* = 20) and ccRCC tissues (*n* = 20). (**H**, **I**) Flow cytometry was used to test CD45RO fluorescence intensity of CD8^+^ T cells (H) and MFI was counted (I). (**J**, **K**) CD45RO protein levels of adjacent normal renal tissues (*n* = 35) and ccRCC tissues (*n* = 35) were tested by western blot and IHC. Means±SEM of experiment performed in triplicates are shown. ^*^P < 0.05; ^**^p < 0.005; ^***^p < 0.005; ns, not significant.

The expression of different CD45 isoforms depends on differentiation and stimulation of cells. In homo sapiens, CD45RO and CD45RA are thought to be memory and naive T lymphocytes in respective. Flow cytometry was utilized to distinguish CD45RO+CD8+ T lymphocytes /CD45RA+ CD8+ T lymphocytes (Figure 3A). The statistical data of CD45RO+CD8+ T lymphocytes and CD45RA+CD8+ T lymphocytes showed that level of CD45RA+CD8+ T lymphocytes in ccRCC patients (*n* = 274) was lower than healthy people (*n* = 350) (*p* = 0.0037) (Figure 3B) and proportion of CD45RO^+^CD8^+^ T cells in ccRCC patients (*n* = 274) was higher than healthy people (*p* = 0.0057) (Figure 3C). Ratio of CD45RO^+^CD8^+^ T lymphocytes /CD45RA^+^ CD8^+^ T lymphocytes was decreased in the circulation of ccRCC patients (*n* = 35, *p* < 0.001) (Figure 3D). Combined with previous results (Figure 2G, 2H, 2I), it was indicated that central memory CD8+ T lymphocytes decrease in ccRCC tissues. To compare CD45RO/CD45RA level in ccRCC with the one in para-carcinoma tissues, we collected pathologically confirmed para-carcinoma tissues and malignant ccRCC tissues for follow-up experiments. The statistical data showed that proportion of CD45RO^+^ CD8^+^ T cells in para-carcinoma tissues were significantly higher than ccRCC (*p* < 0.001) (Figure 3E). In order to further confirm the results, we analyzed *UCHL1* (gene name of CD45RO) of ccRCC through bioinformatics and qRT-PCR. The results showed that both of TCGA data (Figure 3F) and clinical data (Figure 3G) indicated that UCHL1 level reduced in ccRCC tissues compare to para-carcinoma tissues. As for protein level, flow cytometry was utilized to test CD45RO fluorescence intensity of CD8+ T lymphocytes (Figure 3H) and statistical data showed that Median Fluorescence Intensity (MFI) of CD45RO decreased in CD8+ T cells from ccRCC tissues (*p* < 0.0010) (Figure 3I). Western blot (Figure 3J) and IHC (Figure 3K) assays showed that protein level of CD45RO decrease in CD8+ T cells from ccRCC tissues. These results showed that CD45RO+CD8+ T lymphocytes increased in not merely peripheral blood but also the tissues of ccRCC. Both of gene and protein levels of CD45RO increased in ccRCC tissues.

### Although CD45RO+CD8+ T cells inhibit ccRCC progression, they also predict poor prognosis

**Figure 4:**
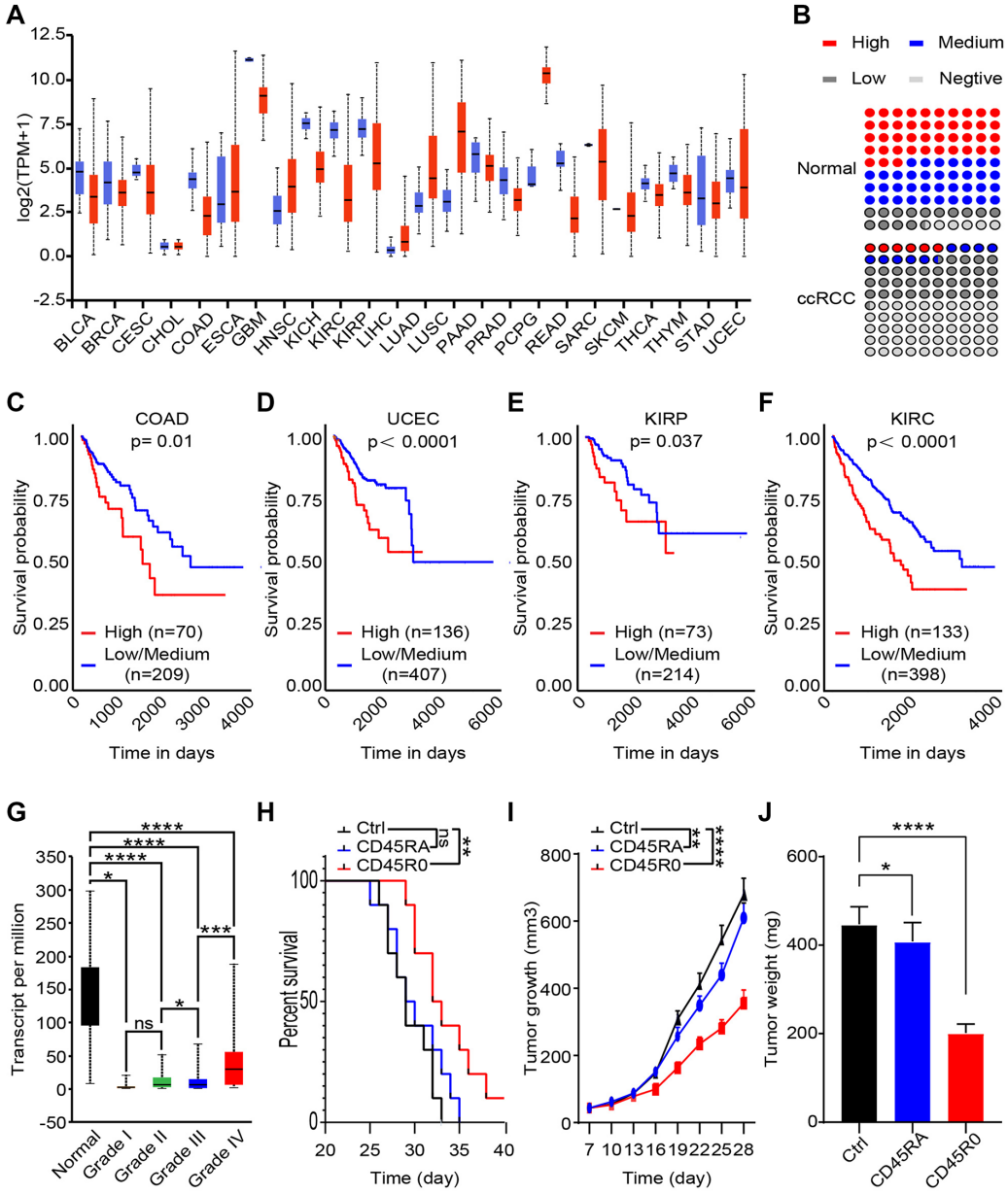
**CD45RO+CD8+ T cells inhibit ccRCC progression, while CD45RO predicts poor prognosis.** CD45RO level from database and clinical specimen were analyzed to explore the potential clinical diagnose and treat value. (**A**) UCHL1 transcript level in 24 cancers tissues compare to normal tissues. (**B**) Statistics of IHC positive rate of CD45RO in ccRCC tissues and normal renal tissues. (**C**, **D**, **E**, **F**) UCHL1 transcript levels of colonic adenocarcinoma (COAD) (C), uterine corpus endometrial carcinoma (UCEC) (D), kidney renal clear cell carcinoma (KIRP) (E) and kidney renal clear cell carcinoma (KIRC) (F). (**G**) UCHL1 transcript levels of normal renal tissues and different degree ccRCC tissues. (**H**, **I**, **J**) We injected 10^8^ CD45RO+CD8+ T cells or CD45RO+CD8+ T cells per week after ccRCC model successfully established. Survival time, tumor growth rate and tumor weight were recorded.

To assess the specificity and universality of UCHL1, we analyzed transcript level of UCHL1 in 24 cancers and corresponding normal tissues. As shown, UCHL1 transcript levels were almost lower in malignant tissues than the normal ones (Figure 4A). As for protein level, we collected 100 para-carcinoma tissues and malignant tissues from ccRCC patients; IHC positive rate of CD45RO in ccRCC tissues (High: 5.8%; Medium: 9.9%; Low: 35.0%; Negative: 49.4%) was lower than normal renal tissues (High: 43.1%; Medium: 37.1%; Low: 14.3%; Negative: 5.4%) (Figure 4B). To further investigate the clinical prognostic role of UCHL1, we analyzed the TCGA data by bioinformatics method. As shown, colonic adenocarcinoma (COAD) (Figure 4C), uterine corpus endometrial carcinoma (UCEC) (Figure 4D), kidney renal papillary cell carcinoma (KIRP) (Figure 4E) and kidney renal clear cell carcinoma (KIRC) (Figure 4F) patients with high UCHL1 levels had a poor prognosis. It indicated that UCHL1 can be used as a potential predictor of tumor prognosis. We further explore the TCGA database and uncloaked that although UCHL1 statistically decreased in ccRCC compare to the normal, it was gradually increasing consistent with tumor grade (Figure 4G). It revealed that though there were more active CD8+ T lymphocytes in advanced ccRCC malignancies, these immune cells were difficult to prevent poor prognosis. To clarify the immune function of CD45RO+CD8+ T lymphocytes and CD45RA+CD8+ T lymphocytes, we injected 1 × 10^8^ CD45RO+CD8+ T lymphocytes or CD45RO+CD8+ T lymphocytes per week after ccRCC model was successfully established. Cancer progression was recorded, and the results showed that both of CD45RO+CD8+ T cells and CD45RA+CD8+ T cells had anti-tumor effect (prolong survival time, limiting tumor growth and reducing tumor weight), while CD45RO+CD8+ T cells treated was more obvious (Figure 4H, 4I, 4J).

This result showed the fact that though immune cells significantly reduced in ccRCC tissues compare to para-carcinoma tissues, amount of CD45RO+CD8+ T cells increased with grade of cancer. CD45RO+CD8+ T cells inhibited ccRCC progression, while in high grade ccRCC tissues, higher CD45RO+CD8+ T cells were difficult to reverse the poor prognosis of patients.

### CD45RO+CD8+T lymphocytes are more proliferative but less apoptotic than CD45RO-CD8+T cells

**Figure 5:**
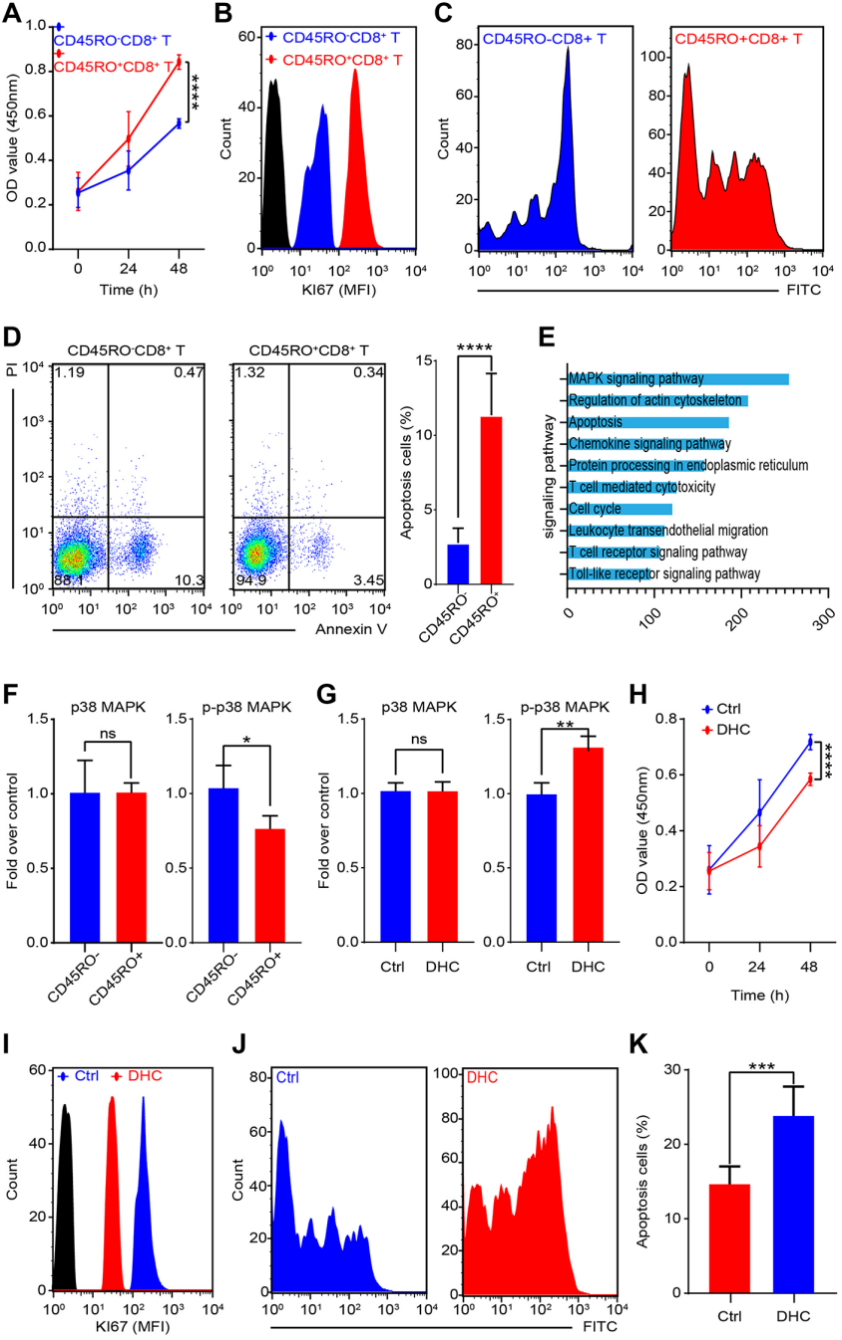
**MAPK signaling promotes proliferation and inhibits apoptosis of CD45RO+CD8+T cells.** CD45RO+CD8+T cells and CD45RO-CD8+T cells were sorted by flow cytometry. (**A**) Cell viability was tested by CCK8 assay at 0, 24 and 48 hours. (**B**) Ki67 fluorescence intensity of CD45RO+CD8+T cells and CD45RO-CD8+T cells were tested by flow cytometry. (**C**) Proliferation rate of CD45RO+CD8+T cells and CD45RO-CD8+T cells were tested by flow cytometry according to changes of CFSE fluorescence intensity. (**D**) Apoptosis status of CD45RO-CD8+T cells and CD45RO+CD8+T cells tested by flow cytometry with PI and Annexin V stanning (right) and apoptosis proportion rates were counted (right). (**E**) Clustering of differential genes between CD45RO-CD8+T cells and CD45RO+CD8+T cells. (**F**) Quantitative analysis of p38/MAPK and p-p38/MAPK of CD45RO+CD8+T cells and CD45RO-CD8+T cells. (**G**) p38/MAPK and p-p38/MAPK of CD45RO+CD8+T cells after dehydrocorydaline chloride treated for 48 hours. (**H**) Cell viability was tested by CCK8 assay at 0, 24 and 48 hours after dehydrocorydaline chloride treated. (**I**) Ki67 fluorescence intensity of CD45RO+CD8+T cells were tested by flow cytometry after treated by dehydrocorydaline chloride treated for 48 hours. (**J**) Proliferation rate of CD45RO+CD8+T cells were tested by flow cytometry according to changes of CFSE fluorescence intensity. (**K**) Proportion of apoptosis CD45RO+CD8+T cells after dehydrocorydaline chloride treated for 48 hours.

To compare differences of CD45RO+CD8+T lymphocytes and CD45RO-CD8+T lymphocytes, we collected PBMCs from circulation of ccRCC patients. CD45RO+CD8+T lymphocytes and CD45RO-CD8+T lymphocytes were sorted by flow cytometry and prepared for following experiments. Proliferation rates of CD45RO+CD8+T lymphocytes and CD45RO-CD8+T lymphocytes were tested by CCK8 assay at 0, 24 and 48 hours (Figure 5A). Ki67, a crucial proliferation related protein, can reflect the proliferation rate of cells. As for Ki67 protein level, flow cytometry was utilized to test CD45RO fluorescence intensity of CD45RO+CD8+T lymphocytes and CD45RO-CD8+T lymphocytes (Figure 5B) and changes of CFSE fluorescence intensity showed that proliferation rate of CD45RO+CD8+T lymphocytes was faster than CD45RO-CD8+T lymphocytes (Figure 5C). We combined PI and Annexin V to tested apoptosis status by flow cytometry and the statistics elucidated that apoptosis proportion rates of CD45RO−CD8+T lymphocytes were higher than CD45RO+CD8+T lymphocytes (Figure 5D). These results indicated that in ccRCC immune environment, CD45RO+CD8+T lymphocytes were more proliferative but less apoptotic than CD45RO-CD8+T lymphocytes. To explore detail mechanisms, we analyzed the different gene between CD45RO^+^CD8^+^T lymphocytes and CD45RO^−^CD8^+^T lymphocytes and genes cluster analysis showed that these different gene were enriched in MAPK signaling pathways (Figure 5E). To verify this mechanism, we tested the protein levels of p38/MAPK and p-p38/MAPK and the statistics showed that p-p38/MAPK protein levels were lower in CD45RO^+^CD8^+^T lymphocytes compare to CD45RO^−^CD8^+^T lymphocytes (Figure 5F). Dehydrocorydaline chloride is an activator of MAPK signaling pathway. To test the activation effect, we treated CD45RO+CD8+T lymphocytes with Dehydrocorydaline chloride (500 nM) for 24 hours and tested phosphorylation of p38/MAPK. The results showed that Dehydrocorydaline chloride effectively increased phosphorylation of p38/MAPK (Figure 5G). To evaluate the effect of p38/MAPK on proliferation, CCK8 assay was used to test the viability at 0, 24 and 48 hours. Proliferation rate of CD45RO+CD8+T cells decreased with dehydrocorydaline chloride treatment (Figure 5H). After treated by dehydrocorydaline chloride for 48 hours, Ki67 fluorescence intensity of CD45RO+CD8+T cells was decreased (Figure 5I). CFSE labeled CD45RO+CD8+T lymphocytes were cultured for 96 hours and flow cytometry showed that the Fluorescence decay was faster after Dehydrocorydaline chloride treated (Figure 5J). As for apoptosis, we treated CD45RO^+^CD8^+^T cells with Dehydrocorydaline chloride for 48 hours and tested apoptosis through flow cytometry. The proportion of apoptosis CD45RO^+^CD8^+^T lymphocytes were increased after Dehydrocorydaline chloride treated (Figure 5K).

### CD45RO+CD8+ T cells effectively inhibit ccRCC progression

**Figure 6:**
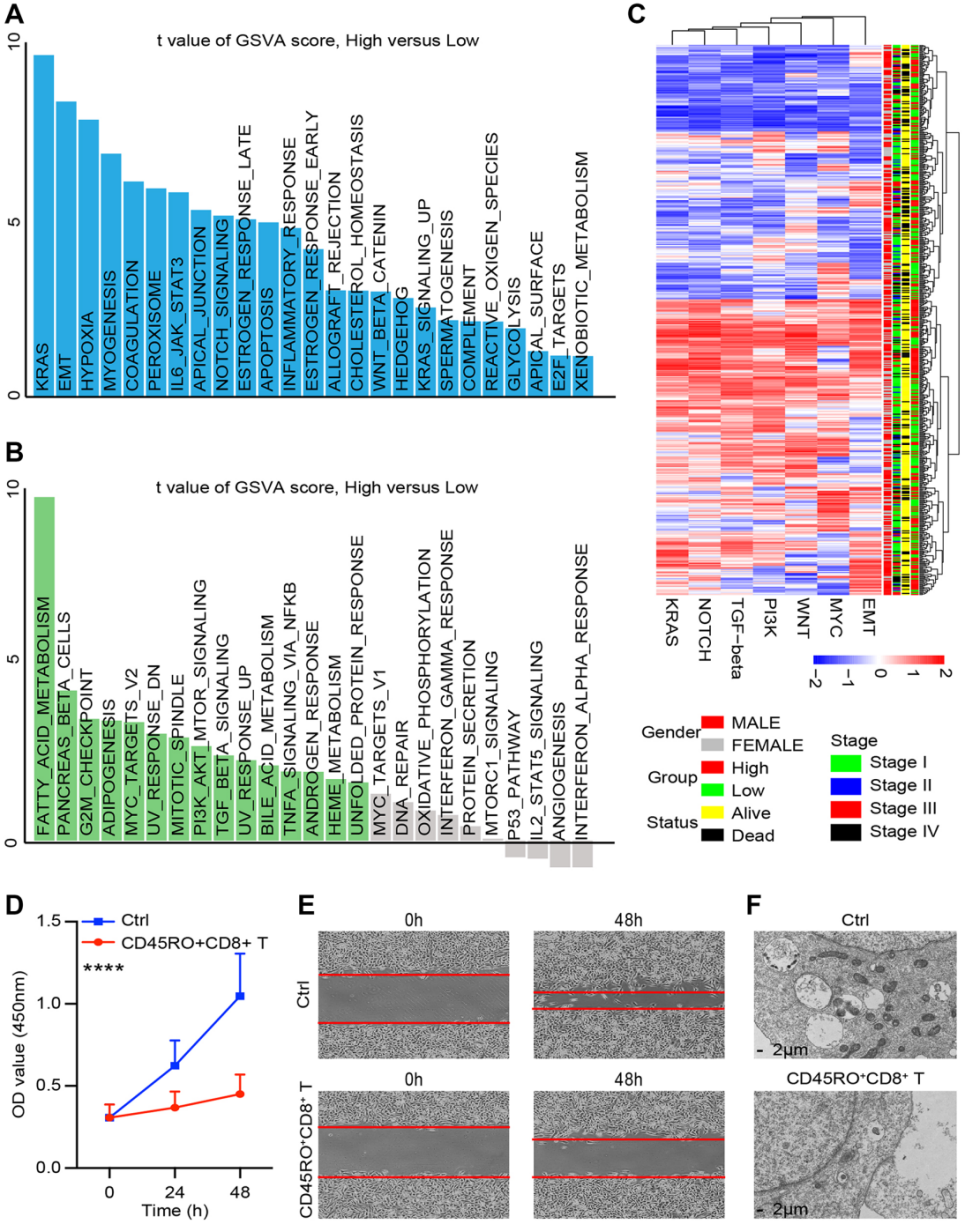
**CD45RO+CD8+ T cells effectively inhibit ccRCC progression.** We co-cultured CD45RO+CD8+ T cells with Caki-2 cells for 48hours and Caki-2 cells were collected. (**A**, **B**) Different genes of Caki-2 with/without CD45RO+CD8+ T cells treated were clustering into upregulated pathways (A) and downregulated pathways (B). (**C**) Heatmap of oncogenic DEPs based on ssGSEA score and their clinical relationships from ccRCC patients according to CD45RO level. (**D**) Proliferation ability of Caki-2 cells with/without CD45RO+CD8+ T cells treated. (**E**) Wound healing assay was used to test the invasion ability of Caki-2 cells. (**F**) Electron microscope images of Caki-2 cells.

CD8+CD45+ T cells perform the crucial role in caner immune environment. In order to assess the impact of CD45RO+CD8+ T cells in ccRCC cell lines, we co-cultured Caki-2 with CD45RO+CD8+ T cells for 48 hours, which were pre-stimulated with ccRCC cells for 24 hours. Gene chip was used to test different genes of Caki-2 with/without CD45RO+CD8+ T cells co-cultured. As shown, a According to TCGA database, we divided ccRCC patients into CD45RO+CD8+ T cells co-cultured group and control group. Upregulated genes were enriched in KRAS, EMT and hypoxia related pathways (Figure 6A) while genes enriched in fatty acid metabolism were downregulated, pancancers beta cells and G2M checkpoint related pathways (Figure 6B). We further divided ccRCC patients into CD45RO high group and CD45RO low group. Gene chips of ccRCC tissues showed that CD45RO low group tended to have higher score in EMT, MYC, WNT, PI3K TGF-beta, NOTCH and KRAS, which were closely related to proliferation and metastasis (Figure 6C). According to analysis results, we tested whether CD45RO+CD8+ T cells had an effect on the biological function of cancer cells. CD45RO+CD8+ T lymphocytes were stimulated first with plate-bound αCD3/CD28 for 3 d and then co-cultured with Caki-2 cells. We tested viability of Caki-2 cells at 0, 24 and 48 hours and found that CD45RO+CD8+ T cells inhibited proliferation of Caki-2 cells (Figure 6D). The results showed that CD45RO+CD8+ T inhibited metastasis pathway, such as EMT, we subsequently co-cultured CD45RO+CD8+ T lymphocytes with Caki-2 cells for 48 hours and tested invasion ability by wound healing experiments. The results demonstrated that CD45RO+CD8+ T lymphocytes inhibited migration ability of Caki-2 cells (Figure 6E). The gene chip also showed that metabolism related pathways changed a lot. After co-cultured with CD45RO+CD8+ T cells for 48 hours, Caki-2 cells were obtained and scanned using electron microscope. The pictures showed that Electron microscope restricted autophagy and mitochondrial aggregation (Figure 6F). These results showed that CD45RO+CD8+ T cells inhibit proliferation, apoptosis and metabolism of ccRCC cells through multiple ways.

### CD45RO combined with related proteins is an effectively diagnostic marker for ccRCC

**Figure 7:**
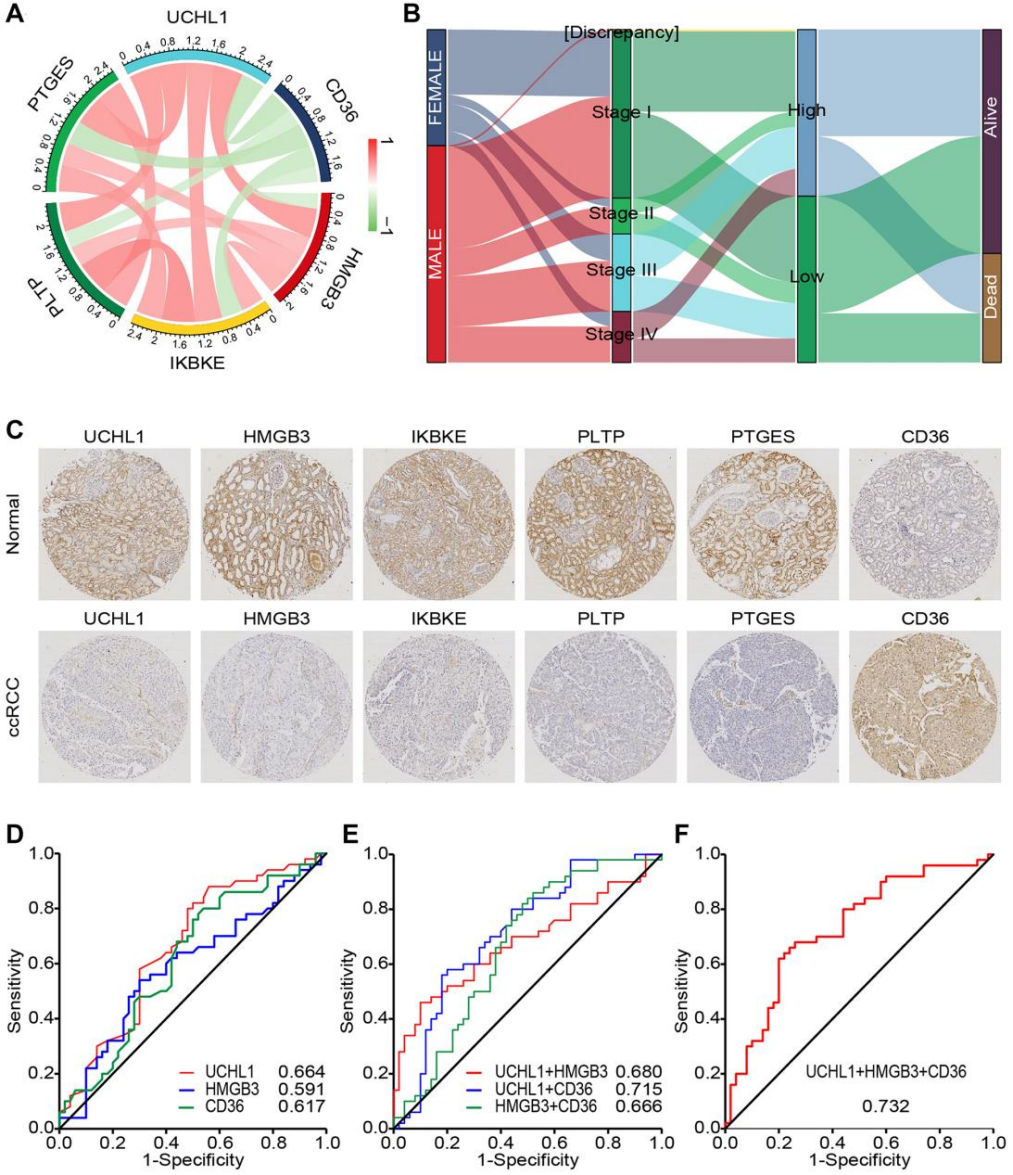
**CD45RO combined with related proteins is an effectively diagnostic marker for ccRCC.** (**A**) The relations between UCHL1 and related genes. Color and line weight represent correlation coefficients. Red: positively related Green: negatively related. (**B**) The correlation and distribution of 2 groups among clinical characteristics in ccRCC patients. Expression level: log2(FPKM+0.01). (**C**) Preventive images of ccRCC tissues and normal renal tissues incubated with UCHL1, CD36, HMGB3, IKBKE, PTLP and PTGES anti-body. (**D**) ROC curve analyses for evaluating the diagnostic potential of UCHL1, CD36 and HMGB3 for ccRCC. (**E**) ROC curve analyses for evaluating the diagnostic potential of UCHL1- CD36, UCHL1 - HMGB3, and HMGB3- CD36 for ccRCC. (**F**) ROC curve analyses for evaluating the diagnostic potential of UCHL1- CD36- HMGB3 for ccRCC.

The role of CD45 does not exist independently but interacts with other genes. To predict these interactions, we analyzed mutual regulation of CD45. Through spearman correlation analysis, UCHL1 was found that it significantly correlated with immune function related molecules including CD36 (*r* = −0.47), HMGB3 (*r* = 0.52), IKBKE (*p* = 0.51), PTLP (*p* = 0.51) and PTGES (*r* = 0.51) (Figure 7A). To verify these relationships and explore clinical significance, we divided ccRCC patients into UCHL1 high group and UCHL1 low group. Distribution of 2 group didn’t show any differences among gender, clinical stage and vital status of the patients (Figure 7B). IHC assays of 35 ccRCC patients showed that UCHL1, HMGB3, IKBKE, PTLP and PTGES increased in malignant tissues compared to the normal ones, while CD36 decreased (Figure 7C). In accordance with IHC analysis, we found that CD36 and HMGB3 are most relevant to UCHL1. Thus, we chose UCHL1, CD36 and HMGB3 as indicators and divided ccRCC patients into high and low UCHL1, CD36 and HMGB3 groups. Receiver operating characteristic (ROC) analysis showed that UCHL1, CD36 and HMGB3 could be distinguishing between patients bearing ccRCC and the healthy ones. There were higher diagnostic accuracy for ccRCC, with measuring the area under the curve (AUC), for diagnosing ccRCC (Figure 7D). Moreover, combination indicators, UCHL1- CD36, UCHL1 - HMGB3, and HMGB3- CD36 had better forecast effectiveness than single indicator (Figure 7E). Combination of UCHL1, CD36 and HMGB3 had the best predictive effect (Figure 7F). Taken together, our results suggested that UCHL1 and related protein, CD36 and HMGB3, might be potential diagnostic biomarkers and therapeutic targets for ccRCC.

### Clinical significance of UCHL1 combined with CD36 and HMGB3 in ccRCC

**Figure 8:**
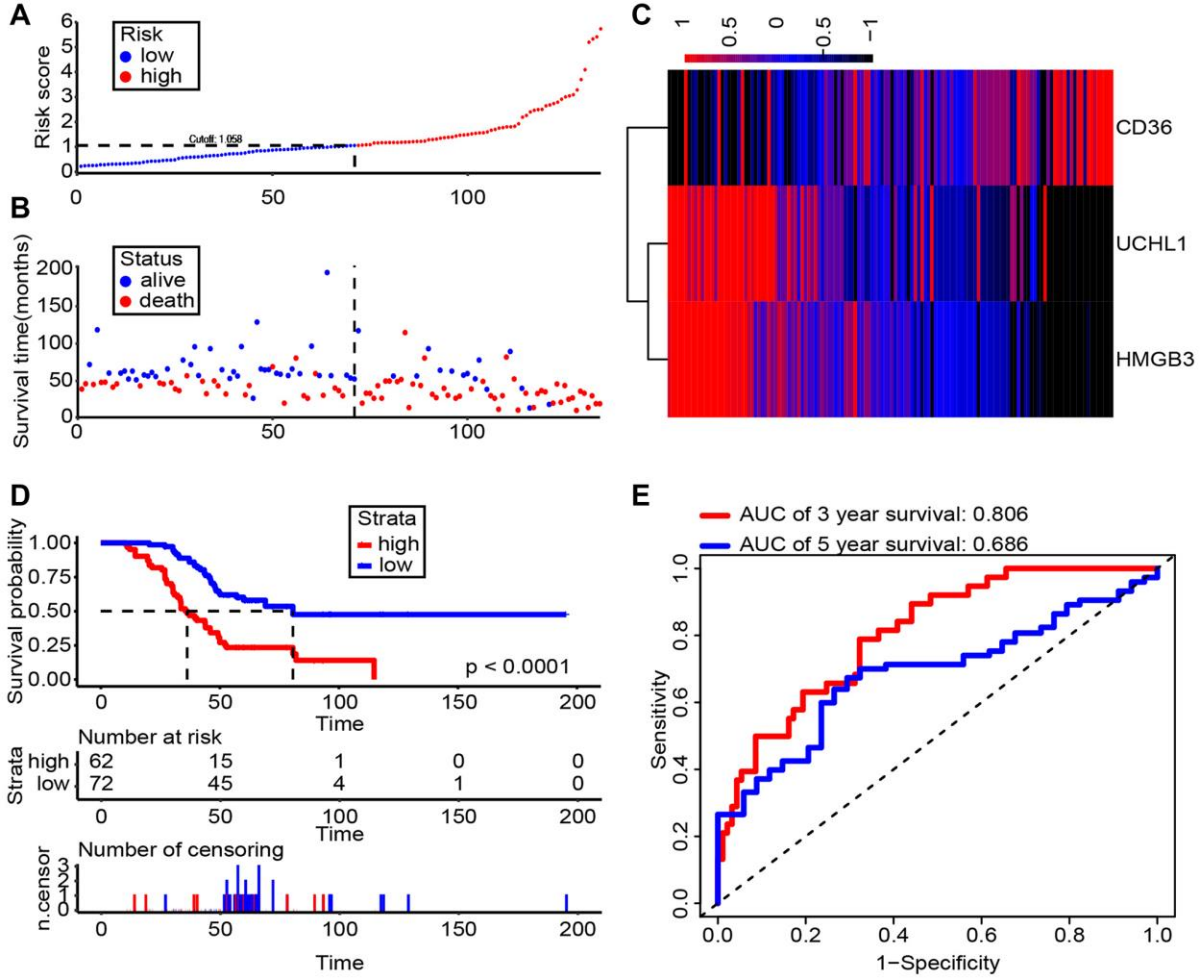
**Risk score analysis of Shanghai General Hospital RCC cohort (*n* = 134).** (**A**, **B**) The risk score and patient’s distribution were visualized with the best cut-off value 1.058. (**C**) Heatmap of RNA expression profiles. Rows represent RNAs and clinical traits while columns represent patients. (**D**) Patients were divided into low-risk (*n* = 72) and high-risk (*n* = 62) groups based on the optimal cut-off point. And Kaplan-Meier method was applied to estimate the survival status. (**E**) To compare the sensitivity and specificity of survival predication, ROC analysis of 3- and 5-year OS was performed based on the risk score.

Accumulating evidence has shown that UCHL1 is recognized as a promising biomarker for prognosis and diagnosis of ccRCC respectively. Therefore, clarifying the clinical significance of UCHL1 and related genes, CD36 and HMGB3, in ccRCC would be of great significance, especially their joint effect. via the risk score generated by multivariate Cox model, we can see patients with higher expression level of CD36, lower expression level of UCHL1 and HMGB3 have poorer survival time (Figure 8A, 8B). The heat map showed that the consistency was good of UCHL1, CD36 and HMGB3 in ccRCC patients (Figure 8C). We further identified the optimal cut-off value (risk score = 1.058) for the classification of two risk groups and a significant differential survival outcome is displayed (Log-rank *P* < 0.0001) (Figure 8D). ROC analysis of 3- and 5-year OS were performed and AUCs were 0.806 and 0.686, respectively (Figure 8E). In general, our results suggested that UCHL1-CD36-HMGB3 could serve as a potential diagnostic biomarker and therapeutic target respectively for ccRCC.

## DISCUSSION

ccRCC has been proved to be a sort of immunogenic tumor associated with various kinds of immune cells [19]. Among them, previous researches demonstrated that CD8+ T lymphocytes played imperative roles in ccRCC pathogenesis while the clinical significance need to be deeply explored [20]. In this study, the immune components of ccRCC were analyzed and we found that CD8+ T lymphocytes had close relationship with tumor progression. Further study indicated that CD45RO+CD8+ T lymphocytes in both circulation and tissues had significant potentials to serve as diagnostic and prognostic biomarkers of ccRCC.

CD8+ T lymphocytes are the major target of tumor immunotherapy [21]. The recent great and swift jumps of cancer immunotherapy, particularly T lymphocytes immune checkpoint blockade (ICB) therapeutics for carcinoma, have unveiled that bunches of CD8+ T lymphocytes as potent immune modulators fighting against advanced malignancies. In an increasing occurrence of cases, CD8+ T lymphocytes have been proved to modulate the regression of large lumpy tumors, leading to durable long-period disease remissions [22]. As for diagnosis and prognosis, high intra-tumoral CD8+ tumor-infiltrating lymphocytes (TILs) proportion was reversely related to tumor volume and could serve as the predicter of improved overall survival of bladder cancer [23]. Likewise, a mammary cancer research illustrated that CD8+TILs status was the most strongly prognostic factor for relapse-free and overall survival [24]. Exploitation of endometrial adenocarcinoma elucidated that CD8+TILs could independently predict the improved survival, particularly for high-risk disease. And intraepithelial CD8+ TILs were reported to serve as a biomarker of efficacy for therapeutic responsiveness in cervical cancer bearing patients suffering radiation and chemotherapy therapy [23]. The reason for this phenomenon perhaps is that the duration of such reactions was fundamentally thought to be based on the capability of T lymphocytes to serve as underlying effectors, followed by generating long-lived cell memory from naïve to mature T cells. This kind of memory has antigen-related specificity and could provide persist host adaptive immune protection. In our analysis, there were no statistical distinction of CD8+ T lymphocytes in circulation between ccRCC patients and the healthy ones, while increasing CD8+TILs in malignant tissues in ccRCC than para-carcinoma tissues. More accurate analysis is necessary to improved predictive accuracy because of complex composition of CD8+ T cells.

CD45 is one of the amplest glycoproteins on the surface of T lymphocytes and is the most thing that we should learn and exploit to discriminate memory T cells and naive T cells [25]. The alternatively spliced isoforms of CD45 are mediated when expressing throughout T lymphocytes advancement and stimulation. In the immune central, naive peripheral T lymphocytes express RA, while stimulated and memory T lymphocytes express RO. To note, the conversion process of RA and RO is interesting. CD45 with α2,6-linked sialic acid and core 1 O-glycans expressed by naive T cells could render it resistant to apoptosis induced by galectin-1 [26]. When activation, CD8+ and CD4+ T lymphocytes express CD45 and CD43 with complex N-glycans and core 2 O-glycans bearing reduced α2,6-linked sialic acid, so that making stimulated T lymphocytes are subjected to apoptosis induced by galectin-1. And it has been reported that, in Hodgkin Lymphoma, the galectin-1 has been illustrated to modulate immune suppressive functionality of CD8+ T lymphocytes, which could support the speculation that the RO/RA ratio descending in patients loading cancer might be ignited by at least the galectin-1 expressed by tumor cells with CD45RO+ T cells eventually sinking into apoptosis yet CD45RA+ T cells [27]. For the specific mechanism, we found that active MAPK signaling pathway in CD45RO T cells is the main reason for higher apoptosis rate. What’s worse, if the proportionality of CD45RA+ T cell escalates, i.e., CD45RO and CD45RA ratio (RO/RA) becomes lower, specific T cells’ life span can be bulked facilitating the impairment of immune defenses, notably in the territory of tumor immune escape, which is in accordance with our results demonstrated above both in clinical and experimental practices CD45RO+ T cells significantly associated with overall survival possibility. Also, Ahmadvand S et al. reported that CD3+, CD8+, Foxp3+ and CD45RO+ TILs in mammary carcinoma serve as prognostic predictors for disease-free survival (DFS) and overall survival (OS). In our study, ratio of RO/RA decreased in ccRCC tissues and CD45RO is a good marker for prognosis prediction. Though CD45RO+ CD8+ T cells inhibited ccRCC progression, it also indicated that tumor cells have increased significantly. The reason of this phenomenon is that tumor antigens can activate effector T cells, they also exert immunosuppressive effects on immunity. At this time, cancer have progressed very rapidly, and anti-tumor immune response had little effect.

On the one side, the specific inhibitory functions of naïve T cells could not be overlooked. As we all familiar with, CD8+ T lymphocytes are main cells of cytotoxicity that provide immune safeguard against cancer cells. Tremendous evidences implicated that the T-lymphocytes repertoire is closely related to the host adaptive immune responsiveness and the advancement of malignant diseases. Intriguingly, incremental expression of Fas in CD8+ T lymphocytes has been studied in patients bearing head and neck carcinoma and melanoma, suggesting that CD8+ T lymphocytes in these patients are prepared for apoptosis [28]. Also, in patients with lung adenocarcinoma, a high proportional expression of Fas in the naïve CD8+ T lymphocytes was observed, which could result in specific apoptosis of CD8+ T preferential apoptosis expressing Fas when they arrived the stage of efficacy by FasL being expressed, such as immune cells, tumor cells, or other cells might cause apoptosis [29].

On the other side, memory or mature CD8+ T cell exhaustion by immunosuppressive cells or cytokines might be tenable. As a matter of fact, the ccRCC tumor immune environment is characterized as immune suppressive, with restraining functions of anti-tumor immune cells while eliciting pro-tumor cells or inhibitory cytokines or chemokines. Myeloid-derived suppressor cells (MDSCs) are the main component of pro-tumor genic leukocytes [30]. MDSCs, under prevailing research, belong to a sort of cluster in phenotypical heterogeneity of colony featured by their capability to inhibit functions of T lymphocytes and NK cell [31]. Their counts are statistically higher in sufferers with ccRCC of all clinical stages associated to that in controls, and their relevant enrichment in other malignant lesions positively corresponds to tumor progression and metastasis. Also, a great many inflammation-inducing cytokines harbor potent pro-tumor ability, comprised of IL-1β, IL-5 and MCP-1. However, these pro-tumor cytokines still exist in the unknown area in RCC as in other malignant types. Ultimately, the memory or mature T lymphocytes end up with exhaustion, which modulate cancer eradication and is implicated by cellular surface markers, including programmed death receptor 1 (PD-1) and B and T lymphocyte attenuator (BTLA) [32].

In the study, we found that CD45RO+/CD8+ T lymphocytes increased in both of circulation and cancer tissues of ccRCC. Our findings provided evidence of an anti-carcinoma mechanism of CD45RO+/CD8+ T lymphocytes in ccRCC. We also elucidated the overexpression of UCHL1 in the malignant tissues of ccRCC, which positively correlated with diagnosis and prognosis of ccRCC when combined with related indicators. Our research not merely identifies the critical anti-tumor mechanisms of CD45RO+/CD8+ T lymphocytes in ccRCC, yet also establishes a non-invasive diagnostic method of potentiality and therapeutic tactics for patients bearing ccRCC. In the future, more accurate analysis is necessary to improved predictive accuracy because of complex composition of CD8+ T lymphocytes.
